# When might we break the rules? A statistical analysis of aesthetics in photographs

**DOI:** 10.1371/journal.pone.0269152

**Published:** 2022-07-01

**Authors:** Justin Wang, Marie A. Lee, Thomas C. M. Lee

**Affiliations:** 1 Department of Statistics, University of California at Davis, Davis, CA, United States of America; 2 Department of Art and Graphic Design, University of the Pacific, Stockton, CA, United States of America; Universita degli Studi di Perugia, ITALY

## Abstract

High-quality photographs often follow certain high-level rules well known to photographers, but some photographs intentionally break these rules. Doing so is usually a matter of artistry and intuition, and the conditions and patterns that allow for rule-breaks are often not well articulated by photographers. This article first applies statistical techniques to help find and evaluate rule-breaking photographs, and then from these photographs discover those patterns that justify their rule-breaking. With this approach, this article discovered some significant patterns that explain why some high-quality photographs successfully break the common photographic rules by positioning the subject in the center or the horizon in the vertical center. These patterns included reflections, leading lines, crossing objects, ambiguous lines, implied lines, thirds line subjects, and busy foregrounds for center horizon photographs, and symmetry, circular-shaped objects, thirds line elements, gestalt, framing, leading lines, and perspective lines for center subject photographs.

## Introduction

High-quality photographs, like other artworks, often adhere to a set of design principles agreed upon by photographers. There are six fundamental principles of design: line, shape, space, texture, light, and color [1]. Applying such principles leads to well-known rules in photography, such as the rule of thirds [2] and the rule of odds [3]. Some high-quality photographs deliberately do not possess the desirable features present in most photographs, purposefully violating photographic design principles and rules. They “break the rules,” so to speak. The breaking of the rules is usually based on feel and intuition, as opposed to relying on specific patterns of conditions to hold. We aimed to solidify concrete conditions and patterns under which the breaking of some of the most well-known photographic rules may occur. To do this, we used a quantitative approach based on statistical and image processing techniques to identify and analyze high-quality rule-breaking photographs.

Our work is closely related to aesthetic quality classification. Aesthetic quality classification, a subfield of computer vision, applies classification models and feature construction to automatically differentiate higher quality photographs from lesser quality ones. Two of the most influential early works in the field were [4, 5]. Both approached the problem by generating a set of features based on properties of photographs and then performing classification on this set of features. A total of 52 candidate features were presented by [4] that made some level of intuitive sense. They then fit a classification model with all of the candidate features, and finally performed model selection, retaining the 15 most “important” features. The authors of [5] interviewed professional photographers to figure out what properties they believed comprised a good photograph. Based on the interviews, they came up with six features to approximate the properties described by the photographers.

Works following [4, 5] aimed to improve the quality of the features used to perform the classification. The authors of [6] first extracted the subject region from a photograph, and then devised features that take advantage of the ability to isolate the subject, such as lighting contrast between subject and background, and color simplicity of the background. The authors of [7–9] constructed features based on high-level describable attributes such as whether an animal is present in the photograph. A model with features all based on color harmony was proposed by [10], while a low-level feature approach was proposed by [11], in which most of the features did not correspond to describable attributes or photographic intuition.

Some works in this field abandon the features approach and use deep learning models, specifically deep convolutional neural networks (DCNNs), to classify the photographs and their pixels directly without using features as an intermediate. Such works include [12–14]. Such approaches achieve a lower classification error, and thus higher predictive power than the past feature-based approaches. However, they come at the cost of being much less interpretable than the feature-based approach, as in general, there is no simple way to map features automatically produced by DCNNs to high level features interpretable by humans.

Most recently, however, severals works have studied the problem of predicting the aesthetic scores of images and at the same time identifying human-interpretable features; e.g., [15–17]. These works have opened the door for a new and important direction in the area of interpretable image ranking. In addition, the authors of [18, 19] provided up-to-date reviews in the areas of visual evaluation and recognition.

### Overview of our work

Here we provide an overview of our analysis, which will follow much more closely to the features-based approach, as interpretability of the features is crucial to our analysis. Our features will be high-level and describable, and will correspond to what can be observed in a photograph by the human eye.

We will use the AVA dataset (more below) in our study. Every photograph in this dataset comes with a rating, from 1 to 10. We shall begin with calculating a set of numerical features for all the photographs in the dataset. We will then use these numerical features, together with the photographs’ corresponding ratings, to fit a statistically interpretable classifier. From this, we will identify a set of features that are statistically significant for predicting the ratings. These features are useful tools in analyzing the rule-breaking photographs. We will also collect those photographs with high ratings but are mis-classified as low-quality. Notice that some of these mis-classified photographs could be rule-breaking photographs.

Next, we will use a human-guided automatic algorithm to systematically review these mis-classified photographs, in hopes of identifying a set of features or patterns that may be related to rule-breaking. This human-guided automatic algorithm is mostly based on known photography rules, but also adapts to different exceptions with the input of an experienced user. More details are given below.

Lastly, we will study and compare the behaviors of those statistically significant features from the classifiers with those from the human-guided algorithm, and obtain our paper’s main results: examples of rule-breaking in photography.

We will focus on identifying and analyzing high-quality photographs that violate two specific important photographic rules. The first rule is that photographs with a visible horizon should not place the horizon right in the center of the photograph. Throughout the paper, we will refer to high-quality photographs that violate this rule as “center horizon photographs.” The second rule is that photographs containing a subject should not place the subject in the center of the photograph. We will refer to high-quality photographs that violate this rule as “center subject photographs.”

As we do not have the permission to re-publish the AVA photographs in this journal, we instead provide the direct url links to those photos that we reference below. These photographs are referenced as Fig AVA1, Fig AVA2, and so on.

## Background and preliminaries

Here we will briefly discuss the HSV color model, which we have used in many of our features. Additionally, many of our features were based on the preliminary steps of subject region extraction and the named color histogram, which we also describe below.

### HSV color model

The HSV color model is an alternate representation of the standard RGB model, which models each pixel of an image as a mixture of red, blue, and green [20, 21]. Under the HSV color model, images are modeled as a collection of 3 matrices—H, S, and V, each of size *n* x *m*, where *n* and *m* represent, respectively, the height and width in pixels of the image. The *H*, or hue matrix, represents the overall color of each pixel along with a circular color spectrum. Corresponding to a circle of colors, values in the hue matrix are integers that range from 0 to 360. The *S*, or saturation matrix represents how saturated or “pure” the color of each pixel is. The values in the *S* matrix range from 0 to 100, with 100 being the most saturated. The *V*, or value matrix represents the brightness of each pixel, and the values also range from 0 to 100, with 100 being the brightest. The elements in each of the three matrices are often standardized to fall in the range [0, 1].

### Saliency map

Two of our features require that the region occupied by the subject, or at the very least important objects, be extracted and identified from photographs. These features are the percentage area occupied by the subject and the lighting contrast between subject and background. To extract the subject region, we used the algorithm Robust Background Detection [22] to obtain what is known as a saliency map. A saliency map is a simplified representation of an image that highlights its salient, or important areas [23]. Our resulting saliency map from Robust Background Detection is an image represented by a *n* x *m* matrix of pixel values ranging from 0 to 255, with 255 being the brightest value. Bright pixel values in a saliency map correspond to important regions in an image (generally the subject itself).

For our two features that require the saliency map, we only need a binary representation. In other words, we only need a representation that identifies whether any given pixel belongs to the subject region. The most straightforward way to binarize a saliency map is to choose a threshold t∈ℤ, such that a given pixel’s binary representation is 1 if the pixel value in the original saliency map exceeds *t*, and 0 if it is less than or equal to *t*. From experimentation, we found that *t* = 100 was a good choice.

### Named color histogram

In image processing, a color histogram is a way to represent the distribution of colors in an image [24]. When constructing a color histogram, the image usually is represented in RBG color space and the *R*, *G*, and *B* matrices are each quantized into bins of size *N*. Each pixel of the image is then assigned to one of *N*^3^ bins, producing a histogram with *N*^3^ bins. Color histograms are a useful tool, but they are not very interpretable at a high level, particularly as a visualization of the colors featured in an image because the resulting bins do not correspond well to the colors known to the human eye. Since we would like to identify high-level patterns among images, we used a modified approach to the color histogram that uses recognizable, or named, colors. We shall call our modified approach the named color histogram.

**Fig 1:**
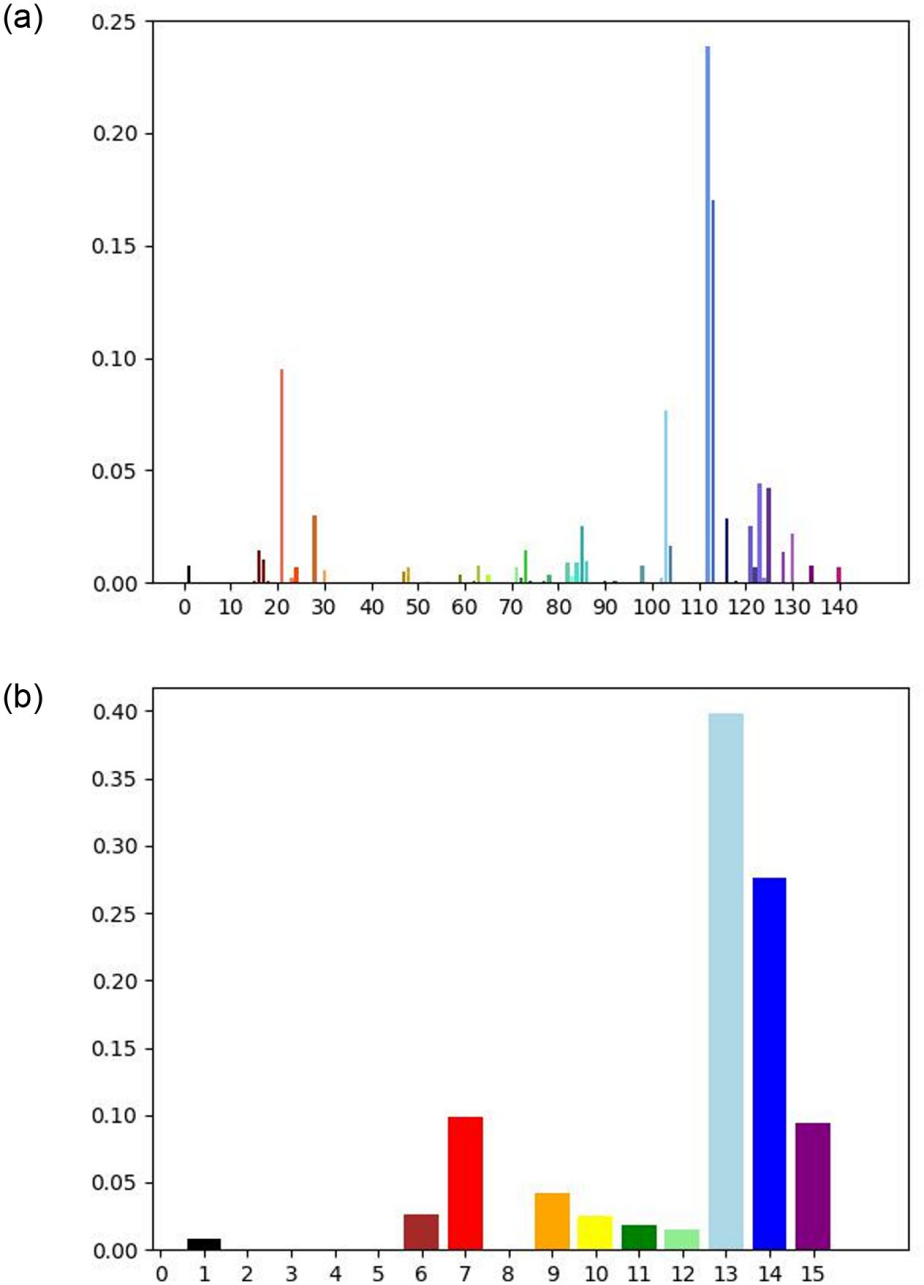
Named colors histogram and a condensed version. (a) Named color histogram. (b) Condensed version.

For our named colors, we used the standard 140 web colors [25]. These are the standard colors used in modern web browsers, and taken together, cover a comprehensive and diverse range of colors. We obtained the coordinate representation in HSV space for each of the 140 colors. To produce a named color histogram for a given photograph, one could then compute the euclidean distance of every pixel in the photograph to each of the 140 named colors, and then assign each pixel to the closest named color. In practice, however, the strategy mentioned above proved to be time inefficient. To speed up the process, we used 8 x 8 non-overlapping sliding windows, averaging the pixel values in each window. We show an example of a named color histogram in Fig 1. In the figure we also provide a condensed histogram that merges similar colors into the same color family. It results in a display of only 16 of the most common named colors.

## Feature description

### Average value and saturation

We start with some simple features: the average value and saturation of each image. High-quality images tend to have a lower value (less bright) and are more saturated (purer in color) than low-quality images. To obtain the average value and saturation of a given photograph, one can simply average all of the values in the *V* and *S* matrices, respectively. More precisely, the average value feature *av* and average saturation feature *as* are given, respectively, by

$$\begin{array}{c} av=\frac{1}{nm}\sum_{i=1}^{n}\sum_{j=1}^{m}v\left(i,j\right)\;\text{and}\;as=\frac{1}{nm}\sum_{i=1}^{n}\sum_{j=1}^{m}s\left(i,j\right), \end{array}$$

where *v*(*i*, *j*) and *s*(*i*, *j*) represent the (*i*, *j*)^th^ element of matrices *V* and *S*, respectively, and *n* and *m* represent respectively the height and width in pixels of the photograph. Note that we are not using average hue as a feature, as it does not make much sense to average all the colors in the color spectrum of the pixels.

### Clarity

One of the most important defining features of high-quality photographs is clarity. High-quality photographs are generally very clear and in-focus, and look very “crisp,” whereas low-quality photographs are often grainy, blurry, and out of focus. We used the clarity feature from [5], which we found to be quite effective.

To define this measure, consider the HSV representation of an image. Let *F* represent the two dimensional Fast Fourier transform of the *V* matrix. Let *C* = {(*u*, *v*):*F*(*u*, *v*)>*ϵ*}, where *ϵ* is a prefixed threshold. Similar to [5], we chose *ϵ* = 5. Our clarity feature *cf* is defined as follows:

$$\begin{array}{c} cf=\frac{\left||C|\right|}{\left||F|\right|}. \end{array}$$

In other words, *cf* represents the percentage of high-frequency elements in *F*.

### Size and aspect

Two simple but very effective features included in our model are size and aspect. High-quality photographs tend to be considerably larger than average than low-quality ones. They also tend to be more concentrated in specific aspect ratios when comparing to low-quality photographs.

Our size feature for a given image is simply *S* = *mn*, where *n* and *m* represent the pixel height and width of the image, respectively. The aspect ratio feature is *A* = *m*/*n*, the width divided by the height. When we construct our model, we will turn aspect ratio into a categorical variable, with its categories defined by aspect ratio ranges.

### Hue count

In the HSV color model, the hue represents actual color identity, whereas saturation and value represent the “pureness” and brightness of that color, respectively. By adjusting the saturation and value, one can obtain a very different “looking” color even when the hue is kept unchanged. Often high-quality photographs do not contain that many different hues. Instead, they vary greatly in saturation and value, resulting in a photograph that appears very rich in color despite the lack of many unique hues. The notable exceptions to this principle are high-quality images that are deliberately colorful, which will indeed have high hue counts. In contrast, low-quality photographs, which often do not have a clear focus, will often have a high hue count. These many different hues are represented in the many different colored objects that are often present in low-quality photographs.

We used the hue count feature from [5]. The idea of this measure is to group the hues of every pixel in a photograph into bins and then count how many of these bins exceed a certain threshold. We divided the overall hue range into 20 bins, each of size 18. Pixels with a value less than 0.15 or greater than 0.95, and pixels with saturation less than 0.2 were discarded before binning. The reason to do this is that any pixel with a value less than 0.15 will effectively appear black, and any pixel with a value greater than 0.95 will effectively appear white, regardless of its actual hue. Pixels with saturation less than 0.2 generally appear as some shade of grey, no matter the hue.

After discarding some pixels as described above, we grouped each remaining pixel into its appropriate hue bin, and constructed a hue histogram with 20 total bins. Let *M* represent the largest bin count among all 20 bins, and let *α* be a small number between 0 and 1. For our hue count feature we used *α* = 0.05. Our hue count feature *HC* is defined as follows:

$$\begin{array}{c} HC=\#\text{bins}\;\text{with}\;\text{bin}\;\text{count}\;\text{greater}\;\text{than}\;\alpha \text{M}. \end{array}$$

### Color nearest neighbors

We devised a measure to assess whether a given photograph more closely resembles a high-quality photograph or a low-quality photograph, in terms of the colors present in the photograph. Using the color histograms of the photographs, we define a color distance measure between two photographs *P*_1_ and *P*_2_ as follows:

$$\begin{array}{c} cd=\sum_{i=1}^{B}{\left(h_{i1}-h_{i2}\right)}^{2}, \end{array}$$

where *B* = 140 represents the total number of bins in the color histogram, and *h*_*ij*_, *j* = 1, 2 represents the frequency of the *i*^*th*^ bin in photograph *P*_*j*_. Using this distance measure, we compute the *K* nearest neighbors for each photograph using *cd*. Then our color nearest neighbors feature *pc* is the proportion of these *K* nearest neighbors that are high-quality photographs:

$$\begin{array}{c} pc=\frac{1}{K}\sum_{i=1}^{K}I\left(N_{i}\;\text{is}\;\text{a}\;\text{high-quality}\;\text{photograph}\right), \end{array}$$

where *N*_*i*_, *i* = 1, …, 20 represents the *i*^th^ nearest neighbor, and **I**(⋅) is the indicator function. For our color nearest neighbors feature, we chose *K* = 20.

### Percentage area of subject

For this feature, we measure the total area percentage of the photograph taken up by the subject and/or prominent objects in the photograph. To do so, we use the binary saliency map *B* and compute the percentage area as follows:

$$\begin{array}{c} pa=\frac{1}{nm}\sum_{i,j}B\left(i,j\right), \end{array}$$

where *n* and *m* represent the height and width of *B*, respectively, and *B*(*i*, *j*) represents the (*i*, *j*)^th^ pixel of *B*.

### Lighting contrast

One commonly known principle in photography is to make the subject stand out from its background. In high-quality photographs, this often translates to the subject being distinctly brighter than the background. We adapted the lighting contrast measure from [6] to measure the contrast between subject and background. To measure the contrast in lighting between the subject and the background, we used the binary saliency map *B* and the brightness matrix *V*. While we used the same feature definition as [6], we used a much more powerful saliency map to detect the subject, which resulted in our lighting contrast measure being much stronger than their version.

We first define *L*_*s*_ and *L*_*b*_, the average lighting within the subject region and background region, respectively:

$$\begin{array}{c} L_{s}=\frac{1}{n_{s}}\sum_{i,j}V\left(i,j\right)\;I\left(B\left(i,j\right)=1\right)\;\text{and}\;L_{b}=\frac{1}{n_{b}}\sum_{i,j}V\left(i,j\right)\;I\left(B\left(i,j\right)=0\right), \end{array}$$

where *n*_*s*_ and *n*_*b*_ represent the number of elements in *B* that are 1 and 0, respectively, and **I**(⋅) is the indicator function. The lighting contrast is then defined as:

$$\begin{array}{c} lc=\text{log}(\frac{L_{s}}{L_{b}}). \end{array}$$

We take the logarithm to prevent the lighting contrast measure from getting extremely small or large. This issue often arises when the background is completely black or white, which leads to extreme values if the logarithm is not taken.

## The AVA dataset

Our work used the AVA dataset [26] for both our high-quality and low-quality photographs. AVA consists of 255, 508 photographs submitted to DPChallenge.com, a photography contest website. Every photograph uploaded to DPChallenge is submitted to a specifically themed “challenge,” with many diverse themes such as “Plants in B/W,” “The Nature of Time,” and “Light Study.” There are a total of 1, 447 challenges represented among all of the photographs in AVA. Every photograph on DPChallenge was given a 1–10 rating by any other registered user with 10 being the highest. AVA provides, for every photograph in the dataset, a count of the number of users who gave the photograph each of the 10 possible numerical ratings. The number of ratings given to each photograph ranges from 78 to 549, with a mean of 210 ratings given. In order to create specific datasets of high and low-quality photographs, we averaged the rating of every photograph in the dataset and then respectively took the top 5% and bottom 5% of all photographs in AVA in terms of average rating. The resulting high and low-quality datasets each contain 12, 777 photographs.Instructions for downloading the AVA dataset can be obtained from https://github.com/mtobeiyf/ava_downloader/tree/master/AVA_dataset.

### Justifying the use of AVA

Since its creation in 2012, AVA has emerged as the preeminent dataset used in aesthetics quality classification. It is by far the largest publicly available dataset of photographs with aesthetic labels. Many researchers in the field use AVA as their main dataset to evaluate their methodology [12, 14, 27]. While we are using many of the same methodology as in aesthetics quality classification, our primary goal is to analyze rule-breaking high-quality photographs. This raises the natural question of whether AVA is suited for this purpose. We will address three potential concerns with using the AVA dataset for our analysis.

The first potential concern is whether user ratings on a website is an accurate measure for objective quality for a given photograph compared to, for example, a panel of photography experts. According to [26], each photograph in the dataset received at least 78 ratings, with the average number of ratings being 210. This indicates that the average rating for each photograph is reasonably aggregated, and is not among a small number of people, which would be subject to high variance. In addition, DPChallenge requires registration in order to rate images, and one would expect at least a base level of interest and knowledge on a user’s involvement to rate photographs. Finally, in observing the high-quality dataset that we obtained, we have found the photographs in the dataset to indeed be of high quality.

The second potential concern is whether the average rating of a photograph accurately captures the entire distribution of ratings. After all, a controversial photograph with many low ratings as well as many high ratings could well end up with a similar score to a photograph with mostly average ratings. This issue is addressed in [26], and they write that the score distributions are well approximated by Gaussian, with a root mean square error less than 0.06. Overall, it appears that the vast majority of photographs have a consensus in scores.

The third potential concern is whether AVA represents a good sample of all photographs in general, given that it is comprised of samples of themed challenges. Every photograph belonging to the same themed challenge will not be independent of one another. There are 1, 447 themed challenges represented among the photographs in the data, and they cover a wide variety of themes. This means that there is roughly an average of 177 photographs per themed challenge. Looking at the challenges themselves, they cover a vast variety of themes and topics in photography. With this many challenges represented in the dataset, it can be safely assumed that a wide breadth of types of photography are represented. Looking through the high-quality photographs, it was clear that there is a large diversity of types of photographs.

## Cleaning, analyzing, and modeling the data

We now describe the details of the data cleaning and modeling process. We also present our results from the data analysis and classification.

### Creating and cleaning the data

We had nine features altogether, and they were all implemented as described in Section **Feature description** Two of them, percentage area of subject and lighting contrast, were based on the saliency map described in Section **Saliency map**. The color nearest neighbors was based on the named color histogram described in Section **Named color histogram**. We were unable to process a tiny fraction of photographs among both the high and low-quality datasets (less than 0.001% for each dataset). The final features dataset for high and low-quality photographs were of dimension 12, 766 x 9 and 12, 762 x 9, respectively.

Photographs without clear subjects often confused the Robust Background Detection algorithm we used to construct our saliency map. The resulting saliency map would often contain a large majority of values that were equal to or close to 255. We found that photographs whose percentage area of the subject measure was greater than 0.7 almost always had this problem. For such photographs it would not be useful to measure any of the features based on the saliency map. We identified 383 high-quality photographs and 169 low-quality photographs that had a percentage area of subject exceeding 0.7. To minimize the impact of these photographs on the classification results, we imputed the mean of the remaining high-quality and low-quality photographs respectively for each of these three features.

The aspect ratio feature was split into categories and converted to a categorical variable via the creation of several dummy variables. There are standard aspect ratios that photographs often adhere to, and we found that most photographs could be classified as being at or close to one of these standard aspect ratios. We ended up creating six categories for aspect ratio: Tall, 3:4, Square, 4:3, 3:2, and Wide based on cutoff ranges for the numerical aspect ratio value. More details about the specific cutoffs we used to construct these categories can be found in the next subsection.

### Feature comparison between high and low quality datasets

To better understand the high-quality photographs in our data and properties of high-quality photographs in general, we performed some rudimentary data analysis on our high-quality dataset. We first converted the value of every feature in both the high and low-quality datasets into their percentile scores. For example, if the percentile of the value of a particular feature in a photograph is 0.7, it means that this photograph has a greater value for this particular feature than 70% of the rest of the data (among both the high and low-quality datasets). We compute 99% confidence intervals for the mean difference in percentile between the high and low-quality datasets for each feature in Table 1. Notice that all these confidence intervals do *not* contain zero, which indicates that all the features are statistically significant in terms of predicting if a photograph is of high or low-quality. Below we discuss the confidence interval for each feature and the reasoning behind their values.

**Table 1:** Confidence intervals of the difference between high and low-quality datasets for each feature.

Feature	Lower Bound	Upper Bound
Avg. Value	−0.056	−0.037
Avg. Saturation	0.005	0.024
Clarity	0.266	0.283
Size	0.199	0.28
Hue Count	−0.099	−0.081
Color Nearest Neighbors	0.166	0.184
Percentage Area	−0.024	−0.009
Lighting Contrast	0.061	0.076

- *Avg. value.* With high confidence, the mean percentile of average value among high-quality photographs is between 3.7% and 5.6% lower than that of low-quality photographs. The high-quality dataset contains a significant subset of photographs that feature a lot of black colors, such as photographs taken in nighttime and objects on black backgrounds. This subset of photographs lowers the average value (brightness) within the high-quality dataset.
- *Avg. Saturation.* With high confidence, the mean percentile of average saturation among high-quality photographs is between 0.5% and 2.4% higher than that of low-quality photographs. The high-quality dataset features many photographs that are generally richer and deeper in color, while low-quality photographs tend to be fainter in color.
- *Clarity.* With high confidence, the mean percentile of clarity measurement among high-quality photographs is between 26.6% and 28.3% higher than that of low-quality photographs. A higher clarity measure of a photograph implies greater clarity, so this is an unsurprising outcome. Low-quality photographs are frequently blurry and unfocused, and high-quality photographs are generally crisp and focused. Some number of high-quality photographs are blurry and thus have a low clarity measure. However, such photographs are not that common, and they tend to be blurry on purpose. The reasons for blurriness among high-quality photographs were usually things like fog and motion.
- *Size.* With high confidence, the mean percentile of size among the high-quality dataset is between 19.9% and 28% higher than that of low-quality photographs. We found high-quality photographs to be considerably larger on average than low-quality photographs. Low-quality photographs are often taken with poor equipment (e.g., camera phones), and thus tend to be smaller.
- *Hue count.* With high confidence, the mean percentile of hue count among the high-quality dataset is between 8.1% and 9.9% lower than that of low-quality photographs. Low-quality photographs are often “messy” in color, as they often contain a random assortment of objects with all types of different hues. High-quality photographs are more focused, which often leads to having a lower number of actual hues.
- *Color nearest neighbors.* With high confidence, the mean percentile of size among high-quality photographs is between 16.6% and 18.4% higher than that of low-quality photographs. Since this feature measures the percentage of the *K* = 20 nearest color neighbors of a given photograph that are high-quality, it makes sense that a high-quality photograph would, on average, have more nearest color neighbors that are also of high-quality. This indicates that high-quality photographs are more likely to resemble one another in terms of color distribution, and similarly for low-quality photographs.
- *Percentage area.* With high confidence, the mean percentile of percentage area among high-quality photographs is between 0.9% and 2.4% lower than that of low-quality photographs. While the difference is only slight, it is nevertheless significant. High-quality photographs are generally careful about the amount of photograph area the subject takes up, and having the subject take up a large amount of area is a deliberate choice. On the other hand, low-quality photographs often are snapshots of people or objects taken quite carelessly, and the result is that the subject often takes up more area.
- *Light contrast.* With high confidence, the mean percentile of light contrast among high-quality photographs is between 6.1% and 7.6% higher than that of low-quality photographs. Contrast in lighting between subject and background is a feature of a good photograph. It is something high-quality photographs often possess and low-quality photographs often lack.

**Fig 2:**
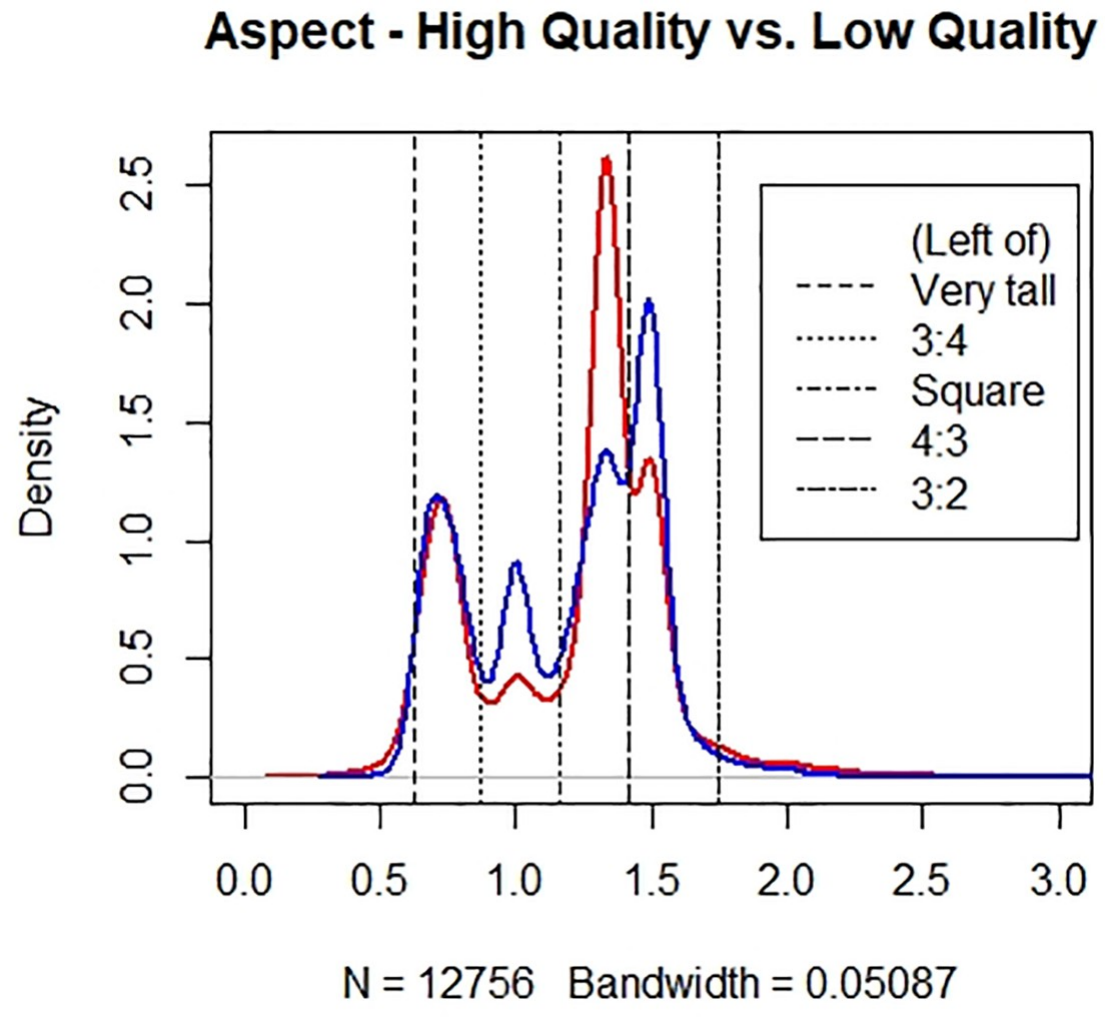
Comparing the distribution of aspect ratio between the high-quality (blue) and low-quality (red) photographs.

To better understand the differences in aspect ratio between high and low-quality photographs, we constructed kernel density plots for the distributions of aspect ratio between high and low-quality photographs in Fig 2. We used a bandwidth of 0.05087 for the plot. The density of high-quality photographs is represented in blue, and the low-quality in red. We grouped the kernel density plot into range categories corresponding to the six aspect ratio categories we created, as depicted in Fig 2. In the legend, a category name that appears next to a given dotted line is the category that comprises the range to the left of that dotted line (but to the right of the previous dotted line, if applicable). For instance, the range between the first and second lines corresponds to a 3:4 aspect ratio. From the figure, we see that there are more low-quality photographs with a 4:3 aspect ratio, but more high-quality photographs with Square and 3:2 aspect ratios. We believe the reason for this is that the 4:3 aspect ratio is the default ratio setting in most non-professional cameras, and thus low-quality photographs, which are often taken with much less care, often just stick to the default ratio. On the other hand, high-quality photographs are a lot more likely to be experimental with different aspect ratios.

### Subcategories

When looking at different categories of photographs, certain design principles and features are more important than others. For example, a photograph of a subject in a completely black background will invariably have the average brightness greatly lowered by the background. Thus, it would be more useful for such photographs to measure the average brightness of the subject instead. Below we describe four subcategories of high-quality photographs we labeled to supplement our analysis. We obtained each subcategory described through a mix of computational methods and manual labeling of the high-quality photographs. For each candidate photograph and each subcategory, we labeled the photograph 1 if it fell into that subcategory, and 0 otherwise.

- *Silhouettes.* A silhouette is the image of a person, animal, object, or scene represented as a solid shape of a single color, usually black, with its edges matching the outline of the subject. They often have a sunrise or sunset in the background and are therefore intensely orange in color, although this is not always the case. Through manual labeling, we identified a total of 842 silhouettes among all of the high-quality photographs. Fig AVA1 in S1 File gives some examples of silhouettes.
- *Landscapes.* We define landscapes as photographs featuring a horizon line, or in which the horizon is at least visible, even if it is not visible as a physical line. The landscape designation will be important in identifying one of our rule-breaks, center horizon. We identified 2902 landscapes among the high-quality photographs through manual labeling. Fig AVA2 in S1 File shows some examples of landscape photographs.
- *Monochrome Background.* We use this designation to refer to photographs that contain a subject with a background of completely one color. We further subdivided this category into six subcategories based on the color of the background. They were as follows: black, white, blue, gray, green, and other. The last category, other, consisted of background colors that did not appear frequently enough to warrant their own category. To obtain monochrome background photographs, we used the saliency map to isolate the background for every photograph in our high-quality data. We then computed the color histogram on the background only. To do this, we used the condensed color histogram mentioned in Section **Named color histogram**, as it provided more precise measurements. We considered a photograph that appeared in more than 90% of a particular color to have that color as a background (occasionally stray colors were picked up slightly in this process, but they had a minimal effect on the final result). We identified a total of 1916 monochrome background photographs among the high-quality dataset. Fig AVA3 in S1 File shows some examples of monochrome background photographs with various background colors.
- *Black and White.* As the name suggests, black and white photographs lack color, and rely only on black, white, and varying shades of grey. From a computational perspective, the *H* and *S* matrices are completely zero for black and white photographs, and thus the only valued matrix is *V*. Equivalently, the *H* and *S* matrices are completely zero if and only if the hue count feature is 0. Thus we obtained black and white high-quality photographs simply by identifying the high-quality photographs that had a hue count value of 0. We identified a total of 1201 black and white photographs within the high-quality data.

### Classification results

We used two classifiers: logistic regression [28] and random forest [29]. Logistic regression is a linear classifier and is highly interpretable, as the direction for the coefficient each feature is clearly specified. If we are willing to put parametric assumptions on the error then we are also able to compute a *p*-value for the coefficient of each feature. Random forest, while less interpretable, is a far more powerful and sophisticated classifier that generally performs better than logistic regression. For logistic regression, we chose 0.5 as the cutoff for predicted probabilities: high-quality if exceeding the cutoff, and low-quality otherwise. For random forest, we used 500 bootstrapped trees and three randomly sampled candidate features at each node split.

We used 5-fold cross-validation and averaged the classification error on the test set from each fold. We found these cross-validated classification errors to be 0.2281 and 0.2173 for logistic regression and random forest, respectively. For logistic regression, noted both the direction and *p*-value of the coefficient of each feature. For each of our 9 features, both the direction and significance matched the results provided in the confidence intervals reported in Table 1.

We present the confusion matrix using the cross-validated predictions for both logistic regression and random forest in Tables 2 and 3, respectively. Most notably, the false positive rates are 0.208 and 0.174, while the false negative rates are 0.248 and 0.26, for logistic regression and random forest, respectively. This indicates that for both classifiers, high-quality photographs are more likely to be misclassified as low-quality photographs than the other way around. This is due to low-quality photographs being generally more homogeneous in their feature directions, e.g., they are generally more blurry, have a higher hue count, and higher saturation. While exceptions certainly exist among low-quality photographs, their feature measurements are more often as expected. On the other hand, high-quality photographs have a much broader diversity in feature measurements. For example, a foggy photograph and a colorful photograph will score “poorly” in the blur and hue count features, respectively, but for a good reason. We also note that logistic regression is less likely to misclassify a high-quality photograph as being of low-quality, but more likely to misclassify a low-quality photograph as being of high-quality. Lastly, we note that no classifier is uniformly better than the other, in the sense that there are photographs that are correctly classified by logistic regression while incorrectly classified by random forest, and vice versa. Roughly, about 70% of the misclassified photographs were common between the two classifiers.

**Table 2:** Confusion matrix for logistic regression.

	Predicted Low	Predicted High	Total
**Actual Low**	10103	2659	12762
**Actual High**	3165	9601	12766
**Total**	13268	12260	25528

**Table 3:** Confusion matrix for random forest.

	Predicted Low	Predicted High	Total
**Actual Low**	10538	2224	12762
**Actual High**	3323	9443	12766
**Total**	13861	11667	25528

**Fig 3:**
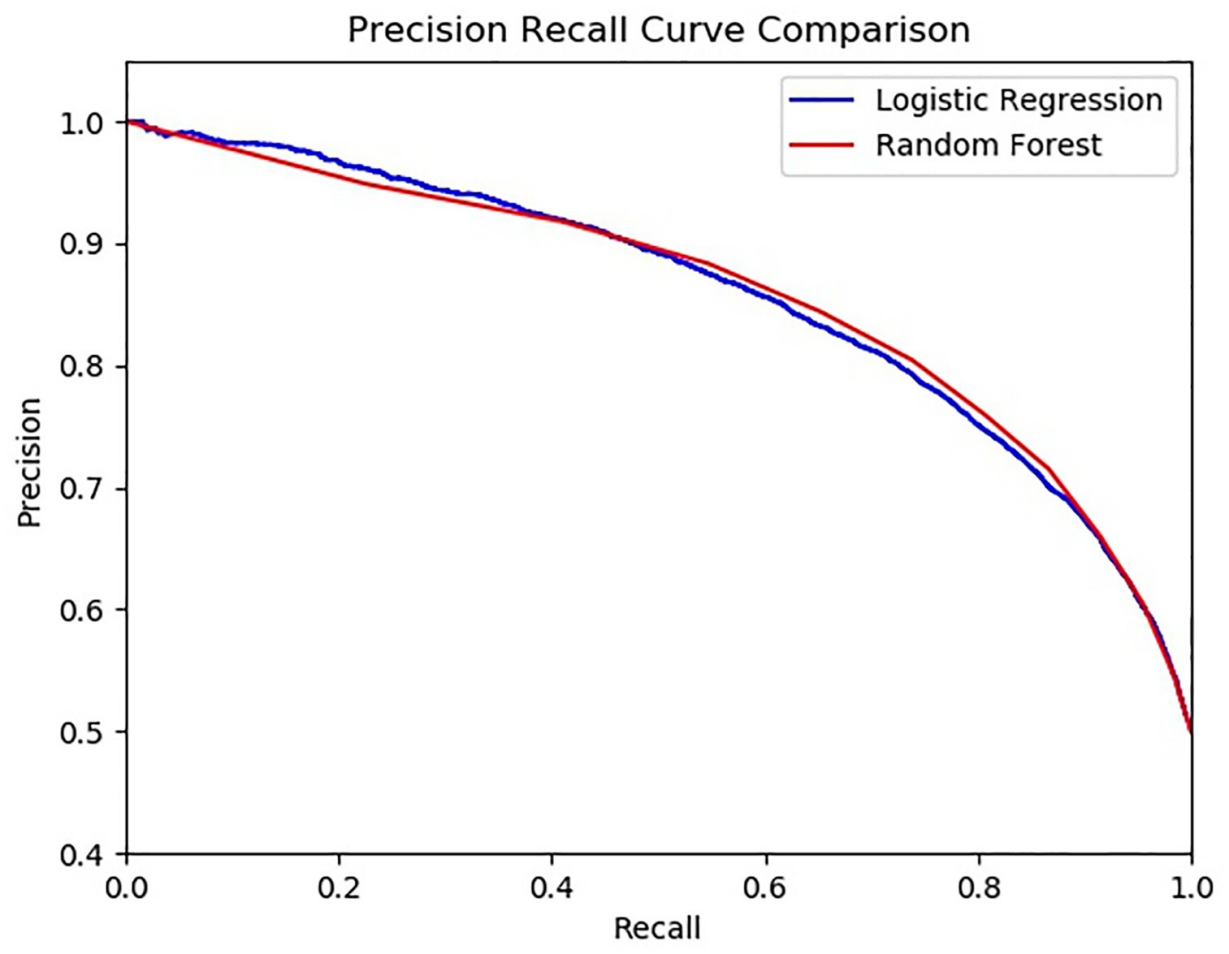
Precision-recall curves for random forest and logistic regression.

We also present the precision-recall curves for both classifiers in Fig 3. It appears that for recall range from 0 to 0.4, logistic regression has higher precision, and for recall range from about 0.5 to 0.9, random forest has higher precision. At recall 0.5, they appear to have about the same precision. The precision and recall associated with our actual logistic regression predictions are 0.783 and 0.752. The precision and recall associated with our actual random forest predictions are 0.809 and 0.74, respectively.

### Human-guided search for rule-breaks

The two classifiers produced good classification results, suggesting that, for most photographs, their qualities can be well predicted by their corresponding feature values. In addition, those correctly-classified high-quality photographs typically are “rule followers,” i.e., they do not break any rules. However, as hinted in Section **Overview of our work**, we are more interested in those high-quality photographs that are mis-classified as low quality. It is because some of these mis-classified photographs could be rule-breaking photographs and potentially led to the discovery of certain rule-breaks.

In general, there are numerous principles and rules that work together to make an artwork successful. Sometimes, a principle can be broken, and yet the artwork is still strong or even stronger. For the current photography problem, typically, there are other principles or rules that take precedence over the one that is broken. By carefully examining those mis-classified high-quality photographs from random forest, we attempted to apply different new combinations of rules and design principles to predict if certain rule-breaks would still lead to high-quality photographs. Then we used human judgment for double-checking the results. We note that our human judgment was based on a co-author who has taught design principles at the college level for over twenty years. While it was a work of an individual reviewer, it was based on rules that have been in use by artists for centuries. Nevertheless, it is conceivable that our human judgment could be improved.

After applying the above procedure, we identified two prominent rules that explain why a photograph was highly rated, yet the classifiers predicted it as low-quality. These two rules are repeated throughout the dataset and can be used to explain when a design principle can be successfully broken; see the next section.

While working on our human-guided algorithm, we discovered other exciting results that are most appealing to designers and photographers. Since understanding such results and the algorithm itself would require a strong background in various design principles, we believe it is more appropriate to present them in a monograph (already in preparation) with designers and photographers as the target audience.

## Analyzing photographic rule-breaks

We identified two specific important photographic rule-breaks found among high-quality photographs. The first is found among landscape photographs and it states that the horizon line should not be placed at the halfway point/center of the photograph. The second is found among photographs containing a subject and it states that the subject should not be placed at the center of the photograph. Both rules are commonly taught to the beginning or intermediate photographers as examples to avoid. However, highly skilled photographers will occasionally choose to break these rules on purpose in their photographs. Traditionally, such rule-breaks are based more on intuition rather than well-specified conditions that allow for breaking the rule. In the following sections, we will explore both rule-breaks computationally using our high-quality dataset, in order to identify common patterns that explain the rule-breaks. We shall refer to these patterns as *explanatory patterns.*

### Horizon lines

Out of 2902 landscape photographs, we identified 307, or 10.6% as having placed the horizon line either precisely at or very close to the center of the photograph. For brevity, we will refer to these photographs as *center horizon photographs*. We identified center horizon photographs among all landscape photographs using the following process:

1. We first used an online machine learning API to estimate the position of the horizon line for a given input landscape photograph. The API consists of a convolutional neural network that was trained on a large number of photographs containing horizon lines. The output was a set of two 2D coordinates, one for each endpoint of the estimated horizon line. In the vast majority of cases, the *y*-coordinate of the two endpoints were the same or very close to being the same, causing the estimated horizon to be a straight or near-straight line.
2. To obtain our horizon line estimate, we averaged the *y*-coordinates of the two endpoints, and then transformed the result to a real number between 0 and 1. This real number represents the relative position of the horizon line in relation to the photograph’s height, with 0 being at exactly the bottom of the photograph, 0.5 being exactly in the middle, and 1 being at exactly the top.
3. Due to truncation errors or some other reasons, occasionally the results obtained from the previous step were not precise. Therefore we performed manual adjustment on the horizon line positions of every landscape photograph to ensure accuracy.
4. After the manual adjustment, we isolated every landscape photograph whose position estimate was between 0.475 and 0.525 and identified them as center horizon photographs.

After obtaining the set of center horizon photographs, we then carefully examined each of them from an artistic point of view, in order to identify explanatory patterns among them. Specifically, we were interested in compositional devices of photographic composition, such that removing these devices would cause the photograph to look strange. Below we describe the eight compositional devices that we identified as the explanatory patterns that justify why these photographs were able to have the horizon line placed in the center.

1. *Reflections across the horizon line (“Reflections”).* Reflections were one of the most common explanatory pattern we found among center horizon photographs. We provide some examples of center horizon photographs featuring reflections in Fig AVA4 in S1 File. Such photographs feature objects reflected across the centered horizon line, almost always over a body of water. Reflections create a symmetry and balance among the top and bottom halves of the photograph, and as such, circumvent the horizon line rule. Reflections are compelling enough on their own that no other explanatory element is generally needed.
2. *Leading lines.* Leading lines are lines in a photograph that lead the eyes to a particular area [1]. Fig AVA5 in S1 File gives some examples of center horizon photographs featuring leading lines. Leading lines generally come in the form of bridges and walkways among the center horizon photographs. They provide depth to the photograph that allows for the viewer to see it in a more three dimensional perspective. This has the effect of mitigating the view of seeing the photograph as “two halves.”
3. *One or more objects “cross” the horizon line “Object Crossing”).* Objects crossings were another one of the most common explanatory patterns that we found among center horizon photographs, after reflections. It refers to having an object or several objects in the center horizon photograph “cross over” the centered horizon line. Fig AVA6 in S1 File gives some examples. Object crossing has the effect of bridging the top and bottom halves of the photograph, so that they no longer appear to be two separate halves.
4. *Horizon line is at least somewhat ambiguous (“Ambiguous Line”).* In some center horizon photographs, the horizon line exists, but is not particularly well defined. Fig AVA7 in S1 File provides some examples. In such photographs, there is much less of a view of a separate top and bottom halves, since their boundary is not quite noticeable or well defined.
5. *There is another implied line or shapes somewhere else (“Implied Line”).* Related to the ambiguity of the horizon line is the notion that there is an implied dividing line other than the horizon line elsewhere in the photograph. This also includes shapes formed by elements such as mountain or forest that appear to create somewhat of a line. Fig AVA8 in S1 File provides some examples of center horizon photographs in which the horizon line is well-defined, but there is another dividing line present in the photograph. When another dividing line not located in the center is present, it breaks the view of a separate top and bottom half.
6. *A subject located on a thirds line (“Thirds Line Subject”).* The rule of thirds is a well-known compositional device that states that important compositional elements of a photograph should generally be placed at thirds intersection points. The rule of thirds is “applied by dividing a medium into thirds both vertically and horizontally, creating an invisible grid of nine rectangles and four intersections” [2]. Some center horizon photographs have a subject, often the main or only subject, located at or near one of the vertical thirds dividing line. Fig AVA9 in S1 File provides an example of such photographs. This element has the effect of providing a thirds balance horizontally, which mitigates the effect of having a top and bottom half.
7. *There is a “busy” foreground that distracts from the horizon line (“Busy Foreground”).* In some center horizon photographs, the bottom half, which represents the foreground of the photograph, commands attention. Fig AVA10 in S1 File provides examples of center horizon photographs with busy foregrounds. Having a busy foreground brings the attention away from the center horizon line and to the foreground, which mitigates the two halves effect.
8. *The sun is present in the photograph that takes attention away (“Sun”).* Some center horizon photographs feature the sun or bright sunlight that distracts from the horizon line. The sun is often located at or near the thirds line, so that it coincides with having a thirds line subject, but this is not always this case. Fig AVA11 in S1 File gives some examples of center horizon photographs featuring the sun.

A total of 82 of the 248 (26.7%) photographs consisted of a reflection across the horizon line. Reflections were compelling enough on their own that no other explanatory pattern was needed alongside them, so we excluded reflection photographs from the subsequent analysis. For the remaining 166 photographs, we tagged each with every applicable compositional element from the above list. The mean and median number of compositional elements exhibited by a center horizon photograph were 2.187 and 2, respectively. The maximum number of compositional elements exhibited by a single center horizon photograph was 4. For each compositional element, we provide in Table 4 the count of center horizon photographs that exhibit that element, as well as the count of center horizon photographs that exhibit *only* that element (we will refer to this as the “exclusive count” of a feature). Exclusive counts for each feature are important to note, as they indicate that for a particular photograph, one compositional element by itself explains the breaking of the center horizon rule. From the table, it appears that leading lines and object crossings are among the most prolific explanatory patterns for breaking the center horizon rule, as they both are commonly exhibited in center horizon photographs, and are the sole explanatory pattern for the rule-break in 4 and 7 of the center horizon photographs, respectively. Sun and busy foregrounds were less so, but also were each the sole justifications for at least some of the center horizon photographs.

**Table 4:** Counts for the center horizon patterns.

Pattern	Count	Exclusive Count
Leading Lines	72	4
Object Crossing	95	7
Ambiguous Line	25	0
Implied Line	51	2
Thirds Line Subject	51	0
Busy Foreground	37	3
Sun	32	1

We used our features described in Section **Feature description** to assess whether there existed any meaningful differences between center and non-center horizon photographs among each of our nine features. We conducted a two-sided *t*-test between measurements of each of our 11 features within center horizon photographs vs. measurements of that feature within non-center horizon photographs. To supplement our *t*-tests, we constructed kernel density plots and side-by-side boxplots to compare the two sets of measurements. The plots were used to identify any possible non-linear differences that the *t*-test would not pick up.

Our analysis found no noteworthy differences between center and non-center horizon photographs among any of our 9 features. For each feature, the p-value of the corresponding *t*-test did not indicate any significant difference. The kernel density plots and side-by-side boxplots also did not suggest any differences, linear or non-linear. We performed a manual inspection of the two sets of photographs and also did not find any differences of note. We also assessed the proportions of black and white and silhouette photographs in both center and non-center horizon photographs. 7.83% of center horizon and 6.76% of non-center horizon photographs were black and white. 18.67% of center horizon and 18.41% of non-center horizon photographs were silhouettes. These proportions were very similar for both subcategories.

### Subject in the center

We identified a total of 225 high-quality photographs that had their subject placed in the center. Among them, 105 were placed in the center in the horizontal direction, but not the vertical direction (“left-right center”). The remaining 120 were placed in the center in both the horizontal and vertical direction (“true center”). Fig AVA12 in S1 File gives an example each of true center photographs. Both types of center placement are considered rule-breaks. To identify true center photographs, we used the following process:

1. We first standardized the values of the positions of each photograph to fall between 0 and 1. It means that, for intance, the center point of any given photograph would always be represented by (0.5, 0.5), the bottom left corner would always be represented by (0, 0), and the top right corner by (1, 1).
2. Next, we computed the weighted centroid of our subject region. To do this, we computed the weighted average of the *x* and *y* coordinates of all non-zero pixels in our saliency map. The weight for each pixel is given by the value of that pixel in the saliency map divided by the max value among all pixels in the saliency map. The resulting centroid is then represented in the standardized notation from the previous step.
3. We then computed the Euclidean distance between the center (0.5, 0.5) of the photograph and the centroid computed in the previous step.
4. We then took every photograph with Euclidean distance less than 0.025 as true center subject photographs.

To identify left-right center photographs, we used a very similar approach to the above. However, instead of computing the Euclidean distance in the third step, we computed the horizontal distance between the center and the centroid, i.e., the absolute difference between 0.5 and the *x*-coordinate of the centroid. We then considered every photograph with a horizontal distance less than 0.025 as left-right center photographs. To prevent misidentification due to potential measurement errors, we then manually inspected each photograph that was identified as being either true center or left-right center, and removed ones that were placed erroneously.

The explanatory patterns present were similar between left-right true center photographs, although they occurred at somewhat different rates. Below we describe seven explanatory patterns:

1. *Symmetry.* Many of the subjects placed in both the left-right and true centers exhibited symmetry. Fig AVA13 in S1 File gives examples of some photographs with centered subjects exhibiting symmetry. While the majority of the exhibited symmetry was from left to right (with a vertical dividing line), there were a few cases of top-bottom symmetry (with a horizontal dividing line), as well as angled symmetry (with a diagonal dividing line). While the majority of cases of symmetry among center subject photographs were quite exact, some photographs exhibited only approximate symmetry.
2. *Circular shaped objects (“Circular”).* Many of the centered subjects had circular shapes, or had a very circular shaped component. Fig AVA14 in S1 File provides some examples of circular shaped objects. Generally speaking, circular shapes lend themselves well to being placed in the center. Oftentimes, but not always, centered subject photographs containing circular shapes also exhibit symmetry.
3. *Important elements in thirds lines (“Thirds Elements”).* Since the subject is often the most important compositional element in the photograph, centered subject photographs still often place important elements placed at “thirds intersection points.” Fig AVA15 in S1 File gives some examples of such photographs.
4. *Gestalt.* Gestalt can be thought of as the principle of “the whole is greater than the sum of its parts” [30]. In other words, in some high-quality photographs, the elements of the photograph are very carefully chosen and highlighted to create an overall “story” to the photograph. Fig AVA16 in S1 File shows examples of centered subject photographs that feature gestalt. In each of these photographs, as well as centered subject photographs featuring gestalt in general, the amount of space on both the left and right sides are vital, as both sides contain elements that add to the overall “story” of the photograph.
5. *Frame around the subject (“Frame”).* Some centered subject photographs featured a framing device around the subject which creates an effect of “frame within a frame” (the outer frame being the photograph itself). Fig AVA17 in S1 File depicts some centered subject photographs with frames around the subject. When such a framing device is present, that frame often acts as the de facto boundaries of the photograph, and thus we would opt to consider the subject’s placement within the framing device rather than the photograph itself. Notably, the frame pictured in a photograph does not have to be an actual frame in the traditional sense, but can be any arrangement that creates a framing-type effect. Frames were also among the most interesting explanatory patterns we found among centered subject photographs.
6. *Leading lines.* Leading lines, discussed earlier in Section **Horizon lines**, also played a role in some center subject photographs. Fig AVA18 in S1 File presents some examples of centered subject photographs featuring leading lines. They feature lines, some blatant and others more subtle that lead either towards or away from the subject (i.e., the center of the photograph). When the leading lines lead away, attention is drawn away from the center, making the subject placement more appropriate.
7. *Perspective lines.* Perspective lines are leading lines that create a different perspective, and often converge towards the horizon. Fig AVA19 in S1 File presents some examples of left-right center subject photographs that had perspective lines. Most often, the subject in such photographs was a person staring straight towards the camera, and the perspective line was a road or walkway leading inwards toward the top of the photograph. Generally, such photographs also exhibited symmetry, as the person and the perspective line itself can both be divided into two halves.

Table 5 summarizes the distribution of the above compositional elements among both left-right and true center subject photographs. Thirds elements, symmetry, and circular objects were the most commonly found explanatory patterns for true center photographs. The latter two patterns are closely related, as many true center photographs featuring circular objects also exhibited symmetry. Among left-right center photographs, symmetry was by far the most common explanatory pattern. Gestalt and thirds elements both also appeared a considerable amount among left-right center photographs. Notably, perspective lines appeared far more commonly in left-right center than true center photographs, but leading lines that were not perspective lines appeared much more commonly in true center photographs. Unlike in Section **Horizon lines**, we will not provide the counts of features that were exhibited exclusively in a given photographs. In contrast to center horizon photographs, the compositional elements found in center subject photographs generally all had enough explanatory power on their own such that no other element was needed.

**Table 5:** Counts for the center subject patterns.

Pattern	True Center Count	Left Right Count
Symmetry	52	63
Circular	40	19
Thirds Elements	53	25
Gestalt	13	25
Frame	10	8
Leading lines	12	0
Perspective lines	5	16

Similarly to Section **Horizon lines**, we assessed whether there exists any meaningful differences between center subject and non-center subject photographs in each feature. We performed two separate analyses, one comparing left-right center subject to non-center subject photographs, and one comparing true center subject to non-center subject photographs. To obtain our non-center subject photographs, we took the set of all high-quality photographs and removed every center subject photograph. It is a good approximation of non-center subject photographs, as the vast majority of high-quality photographs contain at least one object that can be considered a subject. We conducted each of these analyses in the same manner as in Section **Horizon lines**: using *t*-tests, kernel density plots and side-by-side boxplots to compare the two populations of each feature, and then performing a manual inspection of the photographs to identify high-level explanations.

We found no significant linear or non-linear differences between left-right center and non-center subject photographs. When we compared true center to non-center subject photographs, we found that on average true center subject photographs had a significantly higher average *V* value. The *t*-test comparing average *V* between these two groups yielded a *p*-value of 0.0021. The plots for average *V* also confirmed that true center subject photographs had a significantly higher average *V*. Most true center photographs consisted of a subject with monochrome or near-monochrome background. Of the 115 true center photographs, 30 had strictly monochrome backgrounds and 62 had simple backgrounds that were a blend of a few different colors. Such photographs usually had very bright backgrounds, resulting in a higher average *V*. In summary, there were no significant differences among the features in the aggregate between the center and non-center subject photographs, save for a higher average *V* among true center subject photographs.

## Conclusion

With the help of statistical methods, we solidify patterns in photography. Our analysis provided an objective basis for photographers to solidify concepts and ideas that were traditionally based on intuition and feel. More specifically, we discovered some patterns that justify the breaking of the following two common photographic rules:

1. *Center horizon photographs*: photographs with a visible horizon should not place the horizon right in the center of the photograph, and
2. (*center subject photographs*): photographs containing a subject should not place the subject in the center of the photograph.

Patterns we discovered for breaking the first rule included reflections, leading lines, crossing objects, ambiguous lines, implied lines, thirds line subjects, and busy foregrounds. For the second rule, we identified symmetry, circular shaped objects, thirds line elements, gestalt, framing, leading lines, and perspective lines. The most interesting among these patterns were crossing objects and leading lines for center horizon photographs, and gestalt and framing for center subject photographs. These were all phenomena that did not naturally come to mind when imagining what justifications may exist for these specific rule-breaks.

We also devised a statistically interpretable classification model using high-level image features to discriminate between high-quality and low-quality photographs. Performing classification ensured that our features were both effective and discriminative, and established a directional trend for each feature, in terms of being associated with high or low-quality photographs. These features were used to measure whether the center horizon and center subject photographs differed from their respective counterparts. No substantial difference was found for either rule-break, except for the average *V* value in comparing true center subject photographs to non-centered subject photographs.

Future work includes extensions to other types of visual arts, such as paintings, graphics, and digital art. There are many underlying principles that are common to all these different types of visual arts, and a similar analysis may benefit visual artists in various crafts. One could also explore other rule-breaks in photography. While the two that we analyzed were two of the most common, there are other photography rules that could potentially be broken, such as the rule of odds, which states that photographs should generally place an odd number of objects.

## Supporting information

S1 File (PDF)

## References

[1] Webb J. Basics Creative Photography 01: Design Principles. Bloomsbury Publishing; 2017.

[2] Lidwell W, Holden K, Butler J. Universal Principles of Design. Rockport Publishers; 2003.

[3] Ensenberger P. Focus on Composing Photos: Focus on the Fundamentals. Focal Press; 2011.

[4] Datta R, D J, Li J, Wang JZ. Studying Aesthetics in Photographic Images Using a Computational Approach. 9th European Conference on Computer Vision. 2006; p. 288–301.

[5] Ke Y, Tang X, Jing F. The design of high-level features for photo quality assessment. IEEE Computer Society Conference on Computer Vision and Pattern Recognition. 2006; p. 419–426.

[6] Luo Y, Tang X. Photo and video quality evaluation: Focusing on the subject. 10th European Conference on Computer Vision. 2008; p. 386–399.

[7] Luo W, Wang X, Tang X. Content-based photo quality assessment. IEEE Computer Society Conference on Computer Vision. 2011; p. 2206–2213.

[8] Dhar S, Ordonez V, Berg TL. High level describable attributes for predicting aesthetics and interestingness. The 24th IEEE Computer Society Conference on Computer Vision. 2011; p. 1657–1664.

[9] Tang X, Luo W, Wang X. Content-Based Photo Quality Assessment. IEEE Transactions on Multimedia. 2013;15:1930–1943. doi: 10.1109/TMM.2013.2269899

[10] Nishiyama M, Okabe T, Sato I, Sato Y. Aesthetic quality classification of photographs based on color harmony. The 24th IEEE Computer Society Conference on Computer Vision. 2011; p. 33–40.

[11] Marchesotti L, Perronnin F, Larlus D, Csurka G. Assessing the aesthetic quality of photographs using generic image descriptors. IEEE International Conference on Computer Vision. 2011; p. 1784–1791.

[12] Dong Z, Shen X, Li H, Tian X. Photo quality assessment with DCNN that understands image well. 21st International Conference, MMM. 2015; p. 524–535.

[13] Lu X, Lin Z, Jin H, Yang J, Wang JZ. RAPID: rating pictorial aesthetics using deep learning. Proceedings of the ACM International Conference on Multimedia. 2014; p. 457–466.

[14] Jin X, Chi J, Peng S, Tian Y, Ye C, Li X. Deep Image Aesthetics Classification using Inception Modules and Fine-tuning Connected Layer. The 8th International Conference on Wireless Communications and Signal Processing. 2016;.

[15] Reddy GV, Mukherjee S, Thakur M. Measuring photography aesthetics with deep CNNs. IET Image Processing. 2020;14:1561–1570. doi: 10.1049/iet-ipr.2019.1300

[16] Perona FR, Gallego MJF, Callejón JMP. An Application for Aesthetic Quality Assessment in Photography with Interpretability Features. Entropy. 2021;23:1389. doi: 10.3390/e23111389 34828086 PMC8624804

[17] Wu X. Interpretable Aesthetic Analysis Model for Intelligent Photography Guidance Systems. In: 27th International Conference on Intelligent User Interfaces; 2022. p. 661–671.

[18] Bianconi F, Fernández A, Smeraldi F, Pascoletti G. Colour and Texture Descriptors for Visual Recognition: A Historical Overview. Journal of Imaging. 2021;7:245. doi: 10.3390/jimaging7110245 34821876 PMC8622414

[19] Zhang J, Miao Y, Yu J. A Comprehensive Survey on Computational Aesthetic Evaluation of Visual Art Images: Metrics and Challenges. IEEE Access. 2021;9:77164–77187. doi: 10.1109/ACCESS.2021.3083075

[20] Arthur J, Hatt H. The Colorist. D. Van Nostrand Company; 1908.

[21] Smith AR. Color Gamut Transform Pairs. SIGGRAPH 78 Conference Proceedings. 1978; p. 12–19.

[22] Zhu W, Liang S, Wei Y, Sun J. Saliency Optimization from Robust Background Detection. IEEE Conference on Computer Vision and Pattern Recognition. 2014;.

[23] Zhai Y, Mubarak S. Visual Attention Detection in Video Sequences using Spatiotemporal Cues. Proceedings of the 14th ACM International Conference on Multimedia. 2006; p. 815–924.

[24] Gonzalez RC, Woods RE. Digital Image Processing. Prentice Hall; 1977.

[25] Sharma G. Digital Color Imaging Handbook. CRC Press; 2003.

[26] Murray N, Marchesotti L, Perronnin F. AVA: A large-scale database for aesthetic visual analysis. IEEE Conference on Computer Vision and Pattern Recognition. 2012; p. 2408–2415.

[27] Tian X, Dong Z, Yang K, Mei T. Query-Dependent Aesthetic Model With Deep Learning for Photo Quality Assessment. IEEE Transactions on Multimedia. 2015;17:2035–2048. doi: 10.1109/TMM.2015.2479916

[28] McCullagh P, Nelder JA. Generalized Linear Models. 2nd ed. Chapman & Hall; 1989.

[29] Breiman L. Random forests. Machine Learning. 2001;45:5–32.

[30] Koffka K. Principles of Gestalt Psychology. New York: Harcourt, Brace. 1935;.

