# Supplementary material for: When might we break the rules? A statistical analysis of aesthetics in photographs
Source: PLoS One. 2022 Jul 1;17(7):e0269152. doi: 10.1371/journal.pone.0269152 (PMC9249203; doi:10.1371/journal.pone.0269152)
Supplement: S1 File — (PDF) [file pone.0269152.s001.pdf]

https://www.dpchallenge.com/image.php?IMAGE_ID=3416
https://www.dpchallenge.com/image.php?IMAGE_ID=139357
Fig AVA1. Silhouettes.

https://www.dpchallenge.com/image.php?IMAGE_ID=39802
https://www.dpchallenge.com/image.php?IMAGE_ID=558361
Fig AVA2. Landscapes.

https://www.dpchallenge.com/image.php?IMAGE_ID=10366
Fig AVA3. Monochrome background.

https://www.dpchallenge.com/image.php?IMAGE_ID=10838
https://www.dpchallenge.com/image.php?IMAGE_ID=547700
Fig AVA4. Center horizon photographs featuring reflections.

https://www.dpchallenge.com/image.php?IMAGE_ID=664479
Fig AVA5. A center horizon photograph featuring leading lines.

https://www.dpchallenge.com/image.php?IMAGE_ID=233342
Fig AVA6. A center horizon photograph featuring objects crossing the center.

https://www.dpchallenge.com/image.php?IMAGE_ID=833187
Fig AVA7. A center horizon photograph with ambiguous horizon lines.

https://www.dpchallenge.com/image.php?IMAGE_ID=429277
Fig AVA8. A center horizon photograph with implied lines.

https://www.dpchallenge.com/image.php?IMAGE_ID=442842
Fig AVA9. A center horizon photograph with subjects at thirds line intersections.

https://www.dpchallenge.com/image.php?IMAGE_ID=108843
https://www.dpchallenge.com/image.php?IMAGE_ID=233342
Fig AVA10. Center horizon photographs featuring busy foregrounds.

https://www.dpchallenge.com/image.php?IMAGE_ID=221537
https://www.dpchallenge.com/image.php?IMAGE_ID=862189
Fig AVA11. Center horizon photographs featuring sun or sunlight.

https://www.dpchallenge.com/image.php?IMAGE_ID=126461
Fig AVA12. A true center subject photograph.

https://www.dpchallenge.com/image.php?IMAGE_ID=681761
https://www.dpchallenge.com/image.php?IMAGE_ID=1463
Fig AVA13. Center horizon photographs exhibiting symmetry.

https://www.dpchallenge.com/image.php?IMAGE_ID=76259
https://www.dpchallenge.com/image.php?IMAGE_ID=443004
Fig AVA14. Center horizon photographs featuring circular shaped objects.

https://www.dpchallenge.com/image.php?IMAGE_ID=333663
https://www.dpchallenge.com/image.php?IMAGE_ID=518499
Fig AVA15. Center horizon photographs featuring important elements placed in the thirds lines.

https://www.dpchallenge.com/image.php?IMAGE_ID=332250
https://www.dpchallenge.com/image.php?IMAGE_ID=207849
Fig AVA16. Center horizon photographs featuring gestalt.

https://www.dpchallenge.com/image.php?IMAGE_ID=578469

Fig AVA17. A center horizon photograph featuring frames.

https://www.dpchallenge.com/image.php?IMAGE_ID=587311

Fig AVA18. A center horizon photograph featuring leading lines.

https://www.dpchallenge.com/image.php?IMAGE_ID=795208

https://www.dpchallenge.com/image.php?IMAGE_ID=667234

Fig AVA19. Center horizon photographs featuring perspective lines.
